# Spatially Explicit Modelling of the Belgian Major Endurance Event ‘The 100 km Dodentocht’

**DOI:** 10.1371/journal.pone.0164981

**Published:** 2016-10-20

**Authors:** Steffie Van Nieuland, Jan M. Baetens, Bernard De Baets

**Affiliations:** KERMIT, Department of Mathematical Modelling, Statistics and Bioinformatics, Ghent University, Coupure links 653, B-9000 Ghent, Belgium; Peking University, CHINA

## Abstract

‘The 100 km Dodentocht’, which takes place annually and has its start in Bornem, Belgium, is a long distance march where participants have to cover a 100 km trail in at most 24 hours. The approximately 11 000 marchers per edition are tracked by making use of passive radio-frequency-identification (RFID). These tracking data were analyzed to build a spatially explicit marching model that gives insights into the dynamics of the event and allows to evaluate the effect of changes in the starting procedure of the event. For building the model, the empirical distribution functions (edf) of the marching speeds at every section of the trail in between two consecutive checkpoints and of the checkpoints where marchers retire, are determined, taking into account age, gender, and marching speeds at previous sections. These distribution functions are then used to sample the consecutive speeds and retirement, and as such to simulate the times when individual marchers pass by the consecutive checkpoints. We concluded that the data-driven model simulates the event reliably. Furthermore, we tested three scenarios to reduce the crowdiness along the first part of the trail and in this way were able to conclude that either the start should be moved to a location outside the town center where the streets are at least 25% wider, or that the marchers should start in two groups at two different locations, and that these groups should ideally merge at about 20 km after the start. The crowdiness at the start might also be reduced by installing a bottleneck at the start in order to limit the number of marchers that can pass per unit of time. Consequently, the operating hours of the consecutive checkpoints would be longer. The developed framework can likewise be used to analyze and improve the operation of other endurance events if sufficient tracking data are available.

## Introduction

The importance of a healthy life incites people to practice sports. Some are trying to push back their boundaries increasingly further, while others are looking for new challenges. In this context, endurance sports events like (ultra)marathons, triathlons and bicycle races are nowadays becoming very popular. Generally speaking, more and more such events are organized and their number of participants is steadily growing [1–4].

The 100 km Dodentocht, which takes place annually and has its start in Bornem, Belgium, is yet another example of a long distance march. Its marchers have to cover a 100 km trail in at least 10 and at most 24 hours. The first edition took place in 1969 and had only 65 brave youngsters at the start, which clearly contrasts the approximately 11 000 marchers that appeared at the start of the most recent editions. First and foremost, the organizing committee aims at getting people on the move and tries to provide the marchers with a never-to-be-forgotten experience, rather than engaging them in a competition for arriving first at the finish [5].

Using tracking data collected during the editions 2009–2014, the typical dynamics of the 100 km Dodentocht is studied in this paper and a spatially explicit marching model is built to mimic the dynamics of marchers, as such providing a means to the organizers to assess the effect of potential modifications to the starting procedure on the event dynamics. More specifically, this model allows for simulating the speed of every marcher individually along the trail. These speeds can then be used to calculate the passing times of the participating marchers at the consecutive checkpoints in order to allocate the facilities and staff at these checkpoints. To the best of our knowledge, no studies have addressed the facts and figures of endurance events from this point of view.

First, an overview of the tracking data set is given, after which we present its analysis and build and validate the spatially explicit marching model. Finally, we analyze how a modified starting procedure might change the dynamics of the endurance event and formulate guidelines to the organizers for optimizing the first phase of the 100 km Dodentocht.

## Data collection and overview

Like with many other endurance events, the marchers in the 100 km Dodentocht are tracked along the trail by making use of passive radio-frequency-identification (RFID) [6]. More specifically, a battery-less miniature transponder is attached to a hiking boot and gets activated when its carrier steps over an antenna mat that generates a magnetic field and makes the transponder transmit its unique identification number, which is passed to a computer for further processing [7]. Along the 100 km Dodentocht trail, the mats are located only at the entrance of every checkpoint. Once the presence of a 100 km Dodentocht marcher at such a checkpoint has been registered, the time of this registration and the average marching speed between the present and the previous checkpoint is passed in real-time to his/her online profile in order to maximize the involvement of his/her followers [5]. Since there is only one registration per checkpoint, it is important to note that it is impossible to identify the duration of possible resting periods at checkpoints.

Unlike the use of video or GPS technology or the use of health wearables to improve the performances of individual athletes or sports teams [8], RFID data have, to the best of our knowledge, not yet been exploited to gain insight into the dynamics of endurance sports events like long-distance marches, let alone the use of such data to develop a dedicated spatially explicit marching model. This is notable because such an approach could support the organizers in making well-founded decisions on how to improve the experience for their marchers and optimize the allocation of staff and logistics to the different checkpoints along the trail.

The organizing committee of the 100 km Dodentocht provided the RFID data of all marchers who participated in the 100 km Dodentocht marches that were held between 2009 and 2014, as well as the outline of the trail. In addition, the organizing committee provided the gender and birth date of the marchers in the editions 2010–2014. Since no other personal details were registered at the time of registration, characteristics such as overall health condition, fitness level, amount and type of food are not known and their effect cannot be investigated.

**Fig 1:**
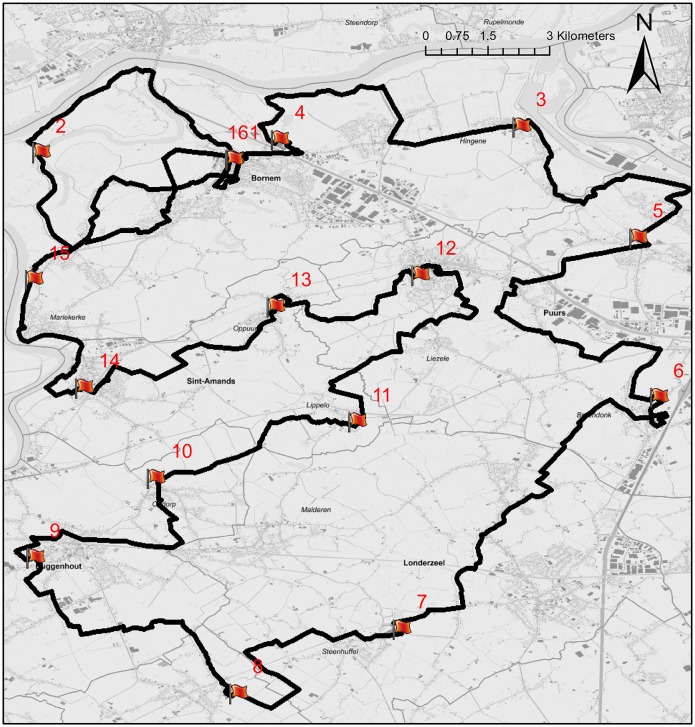
The trail of the 100 km Dodentocht in 2014. The red flags indicate the checkpoints (labeled by their ID) and the thick line indicates the trail itself. (Source: Grootschalig Referentie Bestand Vlaanderen, AGIV.)

Leaving aside minor changes, the trail and the locations of the checkpoints have remained the same over the years covered. As such, we may assume that the influence of the trail configuration did not have an impact on the dynamics of the participating marchers across the subsequent editions. The average distance between the checkpoints and the start is given in Table 1. The start and finish of the 100 km Dodentocht are located in Bornem, Belgium and the loop trail passes through several villages and cities in the provinces of Antwerp, Flemish Brabant and East Flandres (cfr. Fig 1). At every checkpoint, marchers are offered snacks and drinks, while a warm meal is offered at checkpoint 7 (50.0 km). Much-discussed checkpoints are the ones at the breweries of Duvel (checkpoint 6) and Palm (checkpoint 7). Each checkpoint has opening and closing times that are determined on the basis of the minimum and maximum allowed marching speed of about 4.2km h^−1^ and 10km h^−1^, respectively. Marchers arriving before the opening time of a checkpoint have to wait at its entrance until its predefined opening time, while the ones arriving past closure time are disqualified. As a consequence of the imposed marching speed limits, checkpoints at a larger distance from the start have longer opening hours than those located along the first part of the trail and therefore require more operational efforts. In the remainder of this paper, the segment of the trail located in between two checkpoints will be referred to as a section. Every section is assigned an ID, being the number of the checkpoint at the beginning of the section. For every individual marcher, the RFID data was collected until the last recorded checkpoint, *i.e.* the checkpoint at which a marcher is disqualified or finishes. The last recorded checkpoint of marcher *i* is referred to with the symbol *N*_*i*_.

**Table 1:** Average distances along the 100 km Dodentocht trail between the checkpoints and its start in 2009–2014.

Checkpoint ID	1	2	3	4	5	6	7	8	9	10	11	12	13	14	15	16
Distance (km)	1.2	7.5	17.6	24.6	31.7	39.8	50.0	56.2	63.8	69.1	74.2	80.3	84.6	89.7	94.4	100.0

The entire data set contains RFID data of 65 552 marchers, corresponding with 790 230 scans. Across the editions covered in this study, there were on average 22,81% female and thus 77,19% male marchers.

**Fig 2:**
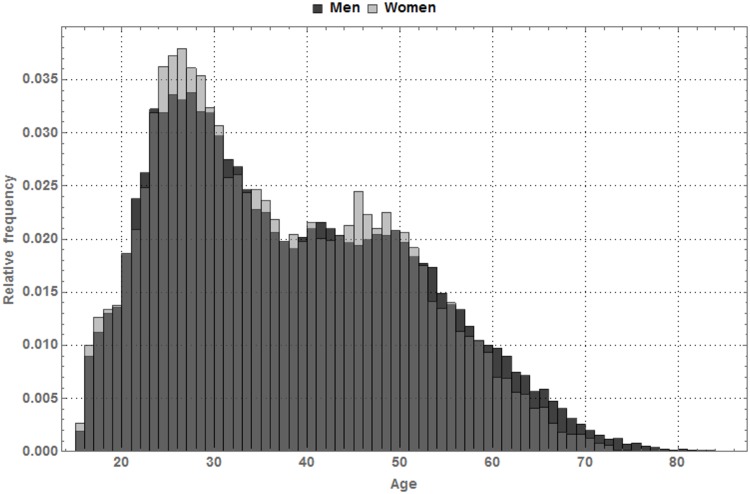
Relative frequencies of the age. Relative frequencies of the age of the marchers (men and women) of the 100 km Dodentocht in 2010–2014.

Fig 2 depicts the relative frequencies of the age of all marchers included in the data set. Marchers between 25 and 30 years old were the most common, while there were fewer marchers aged between 31 and 50 years, though the latter were equally present. Marchers older than 50 and younger than 20 were underrepresented. The relative frequencies were somewhat skewed to the right for male marchers as compared to women, which means that relatively more male marchers were older than 50. The same conclusion can be drawn from the relative frequencies of the individual years, which are not shown for the sake of brevity.

## Data analysis

### Overall statistics

On average, 10 925 marchers showed up at the start of which 61% reached the finish in about 21 h. An overview of the number of marchers, the average marching times and the success rate per edition can be found in Table 2. These numbers are calculated from all the available scans (790 230). In the remainder, however, only marchers with a complete set of scans, *i.e.* a set without missing registrations, are considered. As such, the number of considered marchers is lower than the total number of marchers (cfr. Table 2). As we observed that the overall patterns of the investigated quantities were very similar across the editions 2009–2014, we do not distinguish any further between the different editions in the remainder of this section.

**Table 2:** General statistics of the 100 km Dodentocht in 2009–2014. The number of marchers, the average marching time and success rate per edition and the number of marchers with a complete set of scans.

Edition	# Marchers	Average marching time	Success rate (%)	# Marchers with a complete set of scans
2009	10428	20 h29 min	61.4	10210
2010	10605	20 h22 min	61.4	7441
2011	10507	20 h25 min	58.3	8462
2012	10964	20 h28 min	62.2	9258
2013	11181	20 h30 min	62.4	10237
2014	11867	20 h20 min	58.0	11149

**Fig 3:**
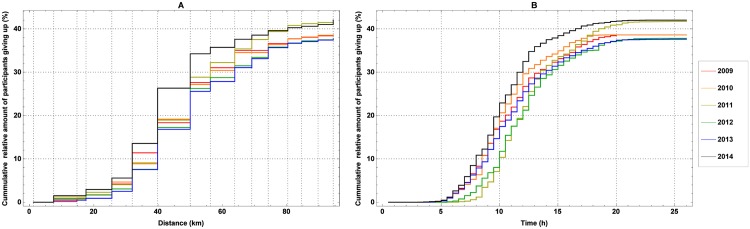
The cumulative relative frequency of marchers retiring. The cumulative relative frequency of marchers retiring versus the distance from the start (a) and the time elapsed since the start (b) during the editions of the 100 km Dodentocht in 2009–2014.

The cumulative relative frequency of men and women retiring as function of the distance from the start and the time elapsed since the start can be found in Fig 3(a) and 3(b), respectively. The chosen bin width for the first one corresponds to the distance between the consecutive checkpoints, while for the latter it is set to 0.5 h. Many marchers retire at checkpoint 3 (17.6 km), and most people quit at checkpoints 5 (31.7 km), 6 (39.8 km) and 7 (50.0 km). A similar conclusion can be drawn from Fig 3(b). Indeed, some marchers are retiring after 3 to 5 hours, but most marchers are retiring after 7 to 12 hours. Once having marched 15 hours, most marchers keep on going.

**Fig 4:**
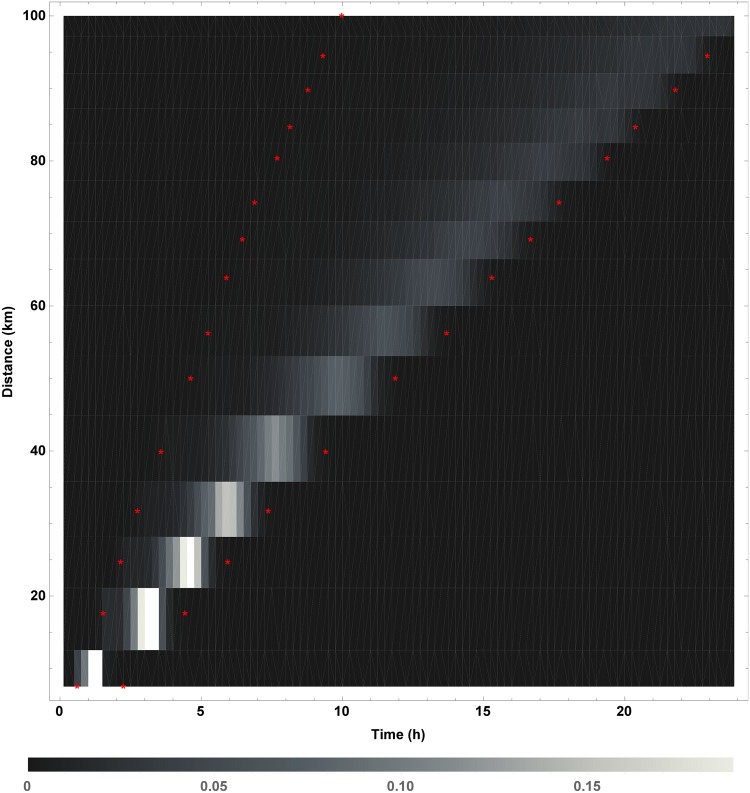
Dispersion of marchers over time and distance. The relative frequency of marchers along the trail versus time elapsed since the start. In red, the opening and closing times of the checkpoints are shown.

The dispersion of all marchers included in the data set along the trail and over time is illustrated in Fig 4. In red, the opening and closing times of the checkpoints are shown. In the beginning of the march, marchers are still clustered, but as they may move at a speed between 4.2km h^−1^ and 10km h^−1^, this clustering becomes much less pronounced as time elapses. This indicates why checkpoints at larger distances from the start require a lot of operational effort. Since most people progress at a relatively low speed, the busiest periods at the checkpoints are typically near their closing times.

**Fig 5:**
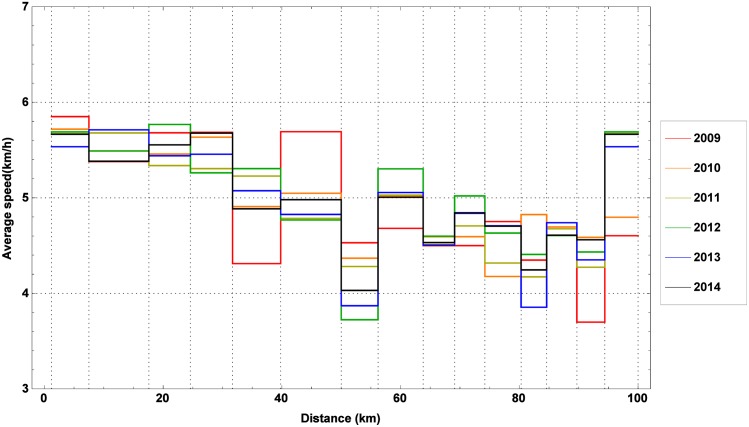
The average marching speed along the trail. The average marching speed along the trail during the editions of the 100 km Dodentocht in 2009–2014.

As a last step of the data analysis, the average marching speed is studied. As can be seen in Fig 5, it appears to decrease steadily along the trail, but there is a pronounced drop noticeable at checkpoint 7 (50.0 km). Yet, given the fact that a warm meal is offered at this checkpoint, this sudden drop is undoubtedly a consequence of relatively long resting periods at this checkpoint, rather than a lower marching speed.

### Correlation analysis

Since it is likely that the speed of a marcher along a given section is related to his/her speed at the preceding and subsequent sections, the autocorrelation between observations separated by *k* sections, with *k* = 1, …, *N*_*i*_−1, is calculated [9]:

$$\begin{array}{c} r_{k}^{i}=\frac{\sum_{s=1}^{N_{i}-k}\left(v_{s}^{i}-{\bar{v}}_{\left(1\right)}^{i}\right)\;\left(v_{s+k}^{i}-{\bar{v}}_{\left(2\right)}^{i}\right)}{{\left[\sum_{s=1}^{N_{i}-k}{\left(v_{s}^{i}-{\bar{v}}_{\left(1\right)}^{i}\right)}^{2}\right]}^{1/2}\;{\left[\sum_{s=k+1}^{N_{i}}{\left(v_{s}^{i}-{\bar{v}}_{\left(2\right)}^{i}\right)}^{2}\right]}^{1/2}}\;, \end{array}$$ (1)

where ${\bar{v}}_{\left(1\right)}^{i}$ and ${\bar{v}}_{\left(2\right)}^{i}$ are the mean of the first *N*_*i*_ − *k* and last *N*_*i*_ − *k* registered marching speeds of marcher *i*, respectively. Positive autocorrelation occurs when the deviation of the speed from the mean tends to be followed by a deviation of the same sign.

**Fig 6:**
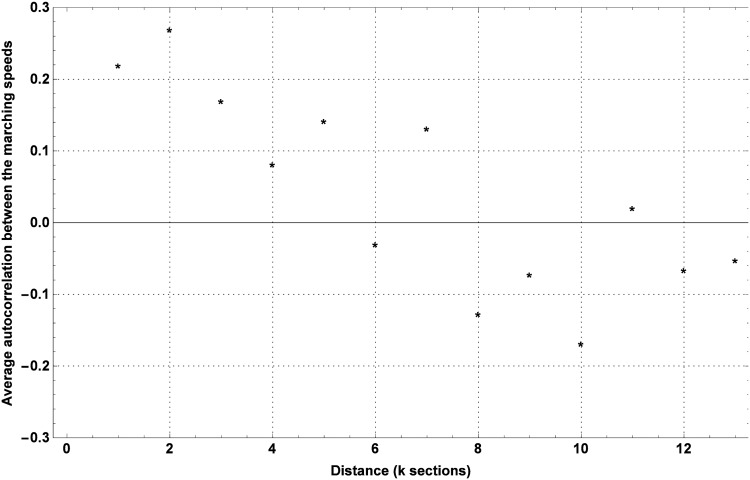
Average autocorrelation between the marching speeds at *k* sections apart.

The average autocorrelation over all marchers and all years is shown in Fig 6. From this figure, it follows that a positive autocorrelation exists between marching speeds up to five sections apart, while a negative one can be seen between marching speeds along sections further apart. The autocorrelation is especially pronounced between marching speeds along two consecutive sections (*k* = 1) and along sections that are two sections apart (*k* = 2), while it is negligibly positive between marching speeds along sections that are four or more sections apart.

**Fig 7:**
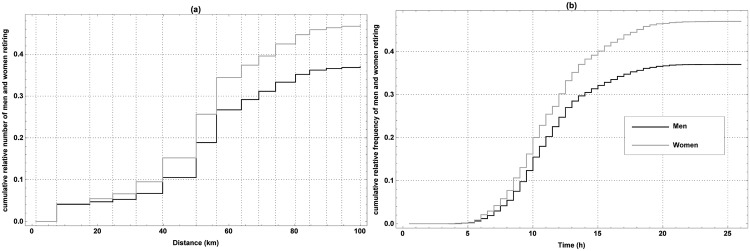
Dynamics of the retires of men and women. The cumulative relative frequency of men and women retiring versus the distance from the start (a) and time since the start (b) of the 100 km Dodentocht in 2010–2014.

**Fig 8:**
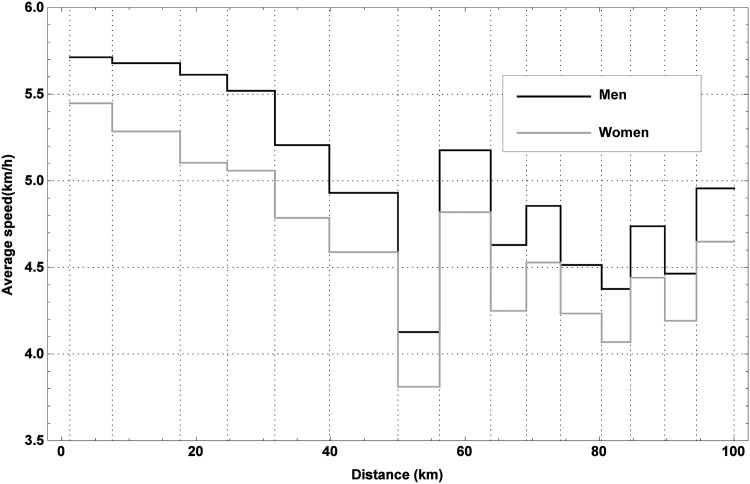
Average marching speed of men and women. The average marching speed of men and women at every section of the 100 km Dodentocht in 2010–2014.

As a next step in the correlation analysis, the influence of age and gender on the marching speed and covered distance is examined. Taking into account that age and gender of the marchers are missing for 2009, only the data of editions 2010–2014 were used for this purpose. First, the effect of gender is considered. In 2010, 21.64% of the marchers were women, while this was 22.54%, 23.28%, 22.45% and 24.16% in 2011, 2012, 2013 and 2014, respectively. Over these editions, the success rate of women was on average 53%, while it was 63% for men. The cumulative relative frequency of men and women retiring as function of the distance from (a) and time since the start (b) can be found in Fig 7, while Fig 8 illustrates the differences between the distributions of the marching speeds of men and women along the trail. Generally speaking, female marchers tend to march somewhat slower than men, while the former also tend to retire at shorter distances from the start and in greater numbers than their male counterparts. As both retiring and marching speed are influenced by gender, this variable can be considered as an important factor. This is also confirmed by a t-test applied to both the observed covered distances and the average marching speeds for men and women.

**Fig 9:**
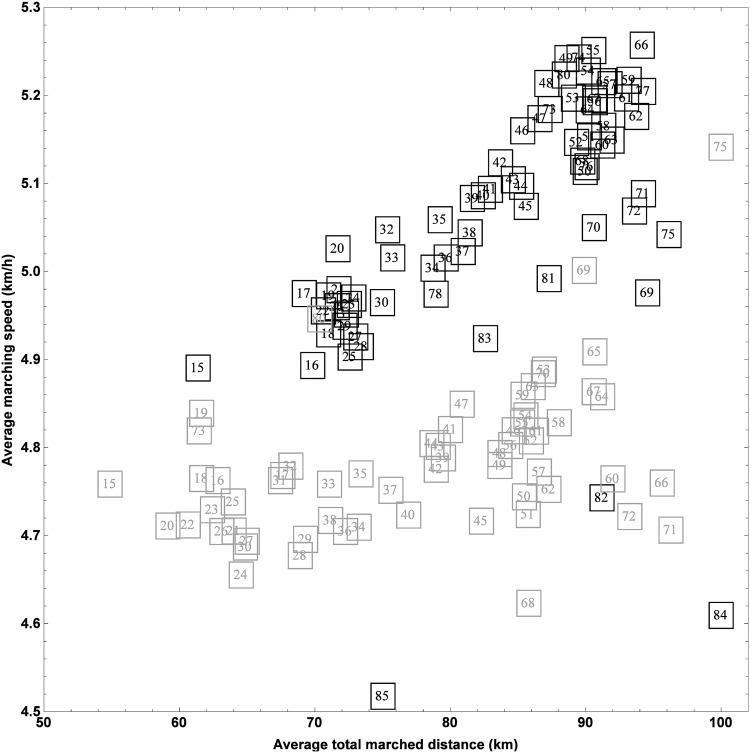
Effect of age on the performance of a 100 km Dodentocht marcher. Each square represents the average total marched distance and the average marching speed for men (black) and women (gray) with the age written down in the center of the square.

The influence of age on the average marched distance and average marching speed is summarized in Fig 9, which depicts the average total marched distance versus the average marching speed per age for men (black) and women (gray). For men, two clusters can be discerned from this plot, a first one containing marchers of at most 30 years old, while the second one contains marchers who are 46 or older. Located between these two clusters are the marchers aged between 32 and 46 who show a steady increase in average marched distance and average marching speed as function of their age. It is remarkable that older marchers tend to walk faster and keep on going longer, though we argue that this might be explained by the fact that younger marchers typically decide to participate in a more impulsive way. Consequently, they appear less prepared at the start of the 100 km Dodentocht than many of the older marchers. For women, age seems to particularly affect the covered distance, while the effect on average speed is less pronounced. Still, a cluster containing relatively younger women (younger than 28), a second cluster containing older women (older than 40), and a transition region are also present in the case of female marchers. Since there is a significant age range where the average marched distance increases as function of the age, a clear-cut clustering of marchers with highly similar age-speed characteristics is not evident.

**Fig 10:**
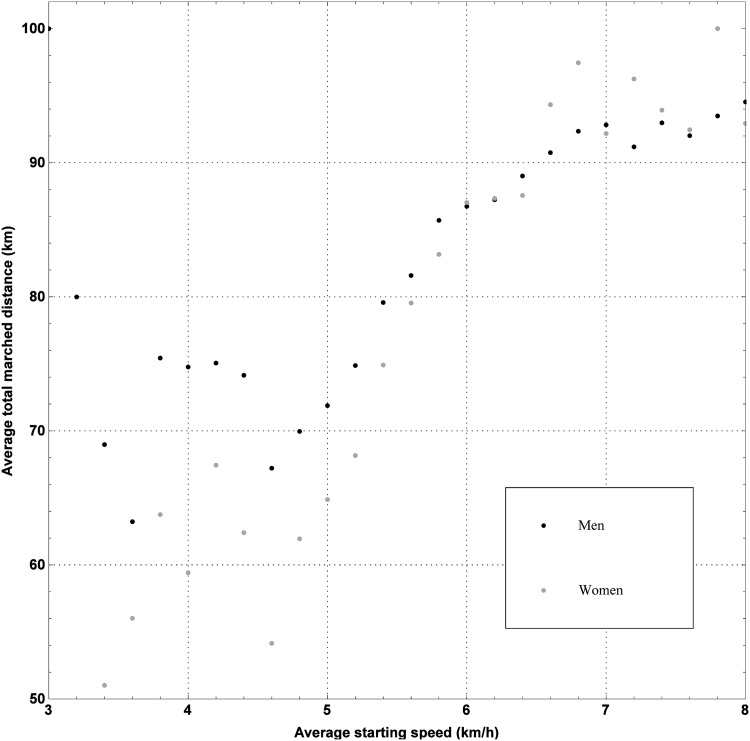
Effect of starting speed on the covered distance of a 100 km Dodentocht marcher. The average total marched distance as a function of the starting speed of a 100 km Dodentocht marcher for men and women.

Finally, the relation between the covered distance and the starting speed is displayed in Fig 10. As can be seen in this figure, the covered distance increases steadily with a higher starting speed, provided the starting speed is higher than 5km h^−1^. Otherwise, this trend is not discernible for men. Nevertheless, the starting speed may serve as an indication of the success rate. In general, it can be stated that marchers starting at a high marching speed cover greater distances.

**Fig 11:**
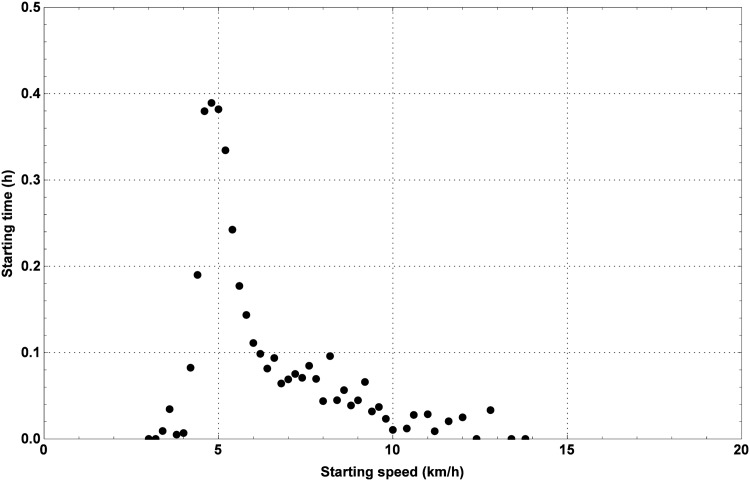
The average time elapsed since the starting signal before the marchers pass by the starting line as a function of the starting speed. The average starting times (men and women) as a function of the starting speed.

After the starting signal of the 100 km Dodentocht has been given, approximately 11 000 marchers start together. As a consequence of the size of the crowd, it takes up to half an hour before the last marcher passes by the starting line. In Fig 11, one can find the average time elapsed since the starting signal before the marchers pass by the starting line versus the starting speed. As one can see, people intending to march fast, will try to start up front.

## A spatially explicit marching model

### Overview

The spatially explicit marching model presented here aims to mimic the speed of an individual in silico marcher along every section of the 100 km Dodentocht trail. Here, we aim at simulating an average edition of the 100 km Dodentocht. Essentially, by using the data from all editions, and hence indirectly accounting for variability, and finally resorting to Monte Carlo simulations, it will be possible to derive confidence intervals that –amongst other things– reflect variations due to external factors.

To grasp the variability in marching speed along a given section *s*, the marching speed along this section *v*_*s*_ has to be conceived as a random variable *V*_*s*_, described by a probability density function (pdf) Ω_*V*_*s*__. Since age A and gender G (cfr. Figs 8 and 9), as well as the marching speed along previous sections P (Fig 6) affect *V*_*s*_, these variables should be taken into account when simulating the dynamics of an individual marcher. More precisely, this means that the speed along the sections under consideration should be sampled from a pdf $\Omega_{V_{s}|\;G,A,P}\left(v_{s}|\;g,a,p\right)$ that is conditioned on G, A and P. Here, G is a binary variable with either man or woman as value, A∈N is a discrete variable, and $P\in R^{+}$ is a continuous variable, which is specified in the Section Model selection. The values under consideration for *V*_*s*_, G, A and P are denoted by *v*_*s*_, *g*, *a* and *p*, respectively.

In addition to the marching speed along every section, the total covered distance by a marcher is estimated. This is done by making use of a hazard function [10]. The hazard function *h*(*n*) describes the conditional probability of retiring at checkpoint *n*, giving that the participant already marched to checkpoint *n*. In order to construct a realistic hazard function, the checkpoint numbers until marchers retire are considered together with the age and gender of marchers, as Figs 7–9 clearly indicate that these individual characteristics are important for understanding the dynamics of retiring. Furthermore, Figs 9 and 10 indicate that there exists a relationship between the marching speed and retiring. As such, the starting speed is also taken into account.

In the remainder of this section, details on the conditional distribution of *V*_*s*_, the exact formulation of P, and the hazard functions are given. This section is concluded by a comprehensive discussion on the selection of the most appropriate model structure.

### Conditional distribution of the marching speed

The conditional distribution $\Omega_{V_{s}|\;G\;,A\;,P}\left(v_{s}|g,a,p\right)$ was constructed experimentally by counting the entries in the data set D fulfilling the conditions on gender, age and marching speed at previous sections. More specifically, the empirical distribution function (edf) ${\hat{F}}_{V_{s}|\;G,A,P}\left(v_{s}|g,a,p\right):R\to \left[0,1\right]$ was determined:

$$\begin{array}{c} {\hat{F}}_{V_{s}|\;G,A,P}\left(v_{s}|\;g,a,p\right)=\frac{\#\{i|v_{s}^{i}\in D\;\wedge \;v_{s}^{i}\leq v_{s}\;\wedge \;G=g\;\wedge \;A=a\;\wedge \;P=p\}}{\#\;\text{of marchers in}\;D\;\text{with}\;G=g\;\wedge \;A=a\;\wedge \;P=p}\;, \end{array}$$ (2)

where $v_{s}^{i}$ is observation *i* of *v*_*s*_ in D. Essentially, the edf ${\hat{F}}_{V_{s}|\;G,A,P}\left(v_{s}|g,a,p\right)$ may be considered as an approximation of the underlying cdf. In contrast to age and gender, the marching speed is a continuous variable, so that a discretization is needed to construct the edf. Here, a bin width of 0.2km h^−1^ was chosen and the speeds were rounded to 0.2km h^−1^.

### Hazard function

The Cox model [11] was used to determine the hazard function for the 100 km Dodentocht. Cox models were determined for both men and women separately, with explanatory variables being age and starting speed. The instantaneous risk of retiring can be written as [11]:

$$\begin{array}{c} \log\left(h\left(n\right)\right)=\log\left(h_{0}\left(n\right)\right)+b_{1}\;a+b_{2}\;v_{1}\;, \end{array}$$ (3)

where *h*(*n*) is the hazard at checkpoint *n*, *a* and *v*_1_ are the values of the explanatory variables age A and starting speed *V*_1_, *h*_0_(*n*) is the baseline hazard, log is the natural logarithm and *b*_1_ and *b*_2_ are model parameters. The coefficients *b*_1_ and *b*_2_ were estimated together with the baseline hazard *h*_0_(*n*) on the basis of the RFID data from the editions 2010–2014. The models were fit using Breslow’s partial likelihood method [12] in Mathematica (Version 9.0.1, Wolfram Research Inc., Champaign, US). The edf of the marchers’ retiring checkpoints based on the cumulative hazard function *h*(*n*) was then used to sample the individual total covered distances using the inverse transform method [13].

### Model simulation

Simulations of average 100 km Dodentocht editions were performed by simulating 10 000 marchers, of which 22.81% were female in accordance with the values reported in the Section ‘Data collection and overview’. Although there were on average 10 925 marchers at the start of the 100 km Dodentocht (cfr. Table 2), we opted to simulate only 10 000 marchers for the ease of interpretability of the simulation results. A simulation generally consists of the following steps. At the beginning of a simulation, every marcher is assigned an age and a starting speed. These values are drawn from the joint probability distribution of A and *V*_1_, which is derived from the RFID data for men and women separately. Subsequently, given an age, gender and starting speed of an in silico marcher, the checkpoint at which he/she retires is derived from the edf of the marchers’ last registered checkpoints. Then, for every in silico marcher individually, the speeds at the consecutive sections are determined up to the one at retiring. These marching speeds are obtained by sampling the edf of *V*_*s*_ given by Eq (3). The sampled checkpoint of retiring and marching speeds are then used to calculate the passing times of marchers at consecutive checkpoints. Since the start takes up to half an hour, the edf of the starting times conditioned on the starting speed *V*_1_ is sampled for a delay and this delay is added to passing times.

The algorithm leading to the simulation of an average 100 km Dodentocht can be found in Algorithm 1.

**Algorithm 1 Pseudo code of the spatially explicit marching model of the 100 km Dodentocht**

• Pseudo code to build the model

Determine the joint edf of A and *V*_1_ for men and women, *i.e.* ${\hat{F}}_{A\;,V_{1}|\;G}\left(a,v_{1}|g\right)$ for all values of *a* ∈ [10, 90] and *v*_1_ ∈ [0, 20], using a discretization step of respectively 1 and 0.2;

**for** *s* = 2 **to** *s* = 15 **do**

 Determine the edf of *V*_*s*_ conditioned on A and P for men and women, *i.e.* ${\hat{F}}_{V_{s}|\;G,A,P}\left(v_{s}|g,a,p\right)$ for all values of *a* ∈ [10, 90] and all values of *v*_*s*_ and *p* ∈ [0, 20], using a discretization step of respectively 1, 0.2 and 0.2 resp.;

 Determine the edf of *V*_*s*_ conditioned on P for men and women, *i.e.* ${\hat{F}}_{V_{s}|\;G,P}\left(v_{s}|g,p\right)$ for all values of *v*_*s*_ and *p* ∈ [0, 20], using a discretization step of 0.2;

 Determine the edf of *V*_*s*_ for men and women, *i.e.* ${\hat{F}}_{V_{s}|\;G}\left(v_{s}|g\right)$ for all values of *v*_*s*_ ∈ [0, 20], using a discretization step of 0.2;

**end for**

Determine the edf of the retiring checkpoints based on the cumulative hazard function including the explanatory variables A and *V*_1_ for men and women separately;

• Pseudo code to simulate marchers

**for all** marchers (For an average 100 km Dodentocht: 10 000) **do**

 Determine the gender *g* of the marcher; (For an average 100 km Dodentocht: 22.81% women)

 Sample *a* and *v*_1_ from ${\hat{F}}_{A\;,V_{1}|\;G}\left(a,v_{1}|g\right)$;

 Sample the retiring checkpoint *n* from the edf of the retiring checkpoints given that A=a, G=g and *V*_1_ = *v*_1_;

 **for** *s* = 2 **to** *s* = *n* − 1 **do**

  **if** (# of marchers in D with G=g∧A=a∧P=p)≠0 **then**

   Sample *v*_*s*_ from ${\hat{F}}_{V_{s}|\;G,A,P}\left(v_{s}|g,a,p\right)$;

  **else**

  **if** (# of marchers in D with G=g∧P=p)≠0 **then**

   Sample *v*_*s*_ from ${\hat{F}}_{V_{s}|\;G,P}\left(v_{s}|g,p\right)$;

  **else**

    Sample *v*_*s*_ from ${\hat{F}}_{V_{s}|\;G}\left(v_{s}|g\right)$;

   **end if**

  **end if**

 **end for**

**end for**

**return** *g*, *a* and *v*_*s*_ with *s* = 1, …, *n* − 1

### Model selection

Based on the autocorrelation between marching speeds at consecutive sections, we can conclude from Fig 6 that the speed along up to the three preceding sections should be taken into account when determining the marching speed along a given section *s*. Still, it should be verified whether a simpler model that is based only on, for instance, the previous marching speed, could be as effective as its counterpart based on the last three registered marching speeds. For that reason, the performance of the spatially explicit marching model was checked for three different forms of the conditional distribution of the marching speed. In the remainder, *M*_1_ is used to refer to the spatially explicit marching model based on a edf involving only *V*_*s*−1_ in addition to age and gender, while *M*_2_ and *M*_3_ are used to refer to the models based upon a edf involving (*V*_*s*−1_ + *V*_*s*−2_)/2 in P and (*V*_*s*−1_ + *V*_*s*−2_ + *V*_*s*−3_)/3 in addition to the age and gender, respectively.

In order to compare the performance of the spatially explicit marching model across the edfs, the models were run 100 times according to the procedure outlined in Algorithm 1.

Besides, the Match distance and the Kolmogorov-Smirnov distance were considered [14–16], two distance functions summarizing the discrepancies between observed values and the values simulated under the model in question [14]. The Match distance quantifies the total difference between the corresponding edfs as follows [14]:

$$\begin{array}{c} d_{M}\left(h,k\right)=\sum_{i}|{\hat{h}}_{i}-{\hat{k}}_{i}|\;, \end{array}$$ (4)

where *h* and *k* are the two edfs under consideration. The Kolmogorov-Smirnov distance only considers the maximum difference between the corresponding edfs [14]:

$$\begin{array}{c} d_{\text{KS}}\left(h,k\right)=\max_{i}|{\hat{h}}_{i}-{\hat{k}}_{i}|\;. \end{array}$$ (5)

**Fig 12:**
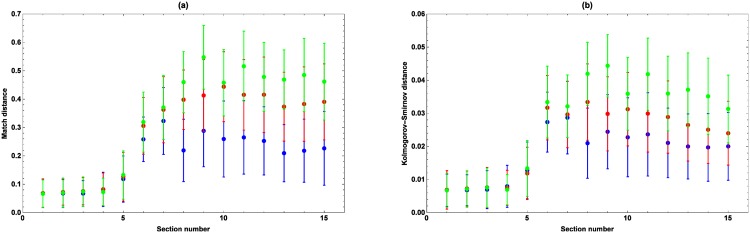
Match (a) and Kolmogorov-Smirnov (b) distances between the edfs of the observed and simulated marching speeds. The Match distance (a) and the Kolmogorov-Smirnov distance (b) for *M*_1_ (blue), *M*_2_ (red) and *M*_3_ (green). The average distances are located in the middle, while the error bars denote twice the standard deviation.

**Fig 13:**
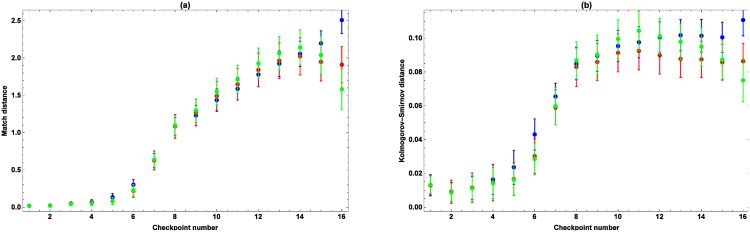
Match (a) and Kolmogorov-Smirnov (b) distances between the edfs of the number of marchers passing by at every checkpoint per 0.25 h. The Match distance (a) and the Kolmogorov-Smirnov distance (b) for *M*_1_ (blue), *M*_2_ (red) and *M*_3_ (green). The average distances are located in the middle, while the error bars denote twice the standard deviation.

The average Match and Kolmogorov-Smirnov distances between the observed and simulated marching speeds and passing times are shown in Figs 12 and 13, respectively. The average distances for the cumulative relative frequency of marchers retiring at every checkpoint can be found in Table 3. From Fig 12 it follows that the distances between the observed and simulated edfs of the marching speeds are low, that they increase from checkpoint 6 on, and that there are differences between the models. More precisely, *M*_1_ is the best at simulating the marching speeds, while *M*_3_ performs the worst. Concerning the passing times, the distances between the observed and simulated number of marchers passing by at every checkpoint per 0.25 h are low if the speeds at the consecutive sections are composed in such a way that the simulated speed at a section is chosen well considering the simulated speeds at all other sections (cfr. Fig 13). For example, a marcher marching fast along the first few sections, is likely to maintain high speeds along the following sections, even if the marcher takes a break along one of these sections. If the model would consistently assign low speeds in this case, these speeds would not match with the previous high speeds, causing a bad composition and high Match and Kolmogorov-Smirnov distances. The distances increase from checkpoint 6 on, with the largest increase at checkpoints 7 and 8, where most people take a break due to the meal that is offered causing a decline of the recorded marching speeds. Because of that, fast marchers taking a break and slower marchers not taking a break have the same marching speeds along the considered sections, which hinders an unambiguous determination of their subsequent marching speeds. For what concerns the passing times, *M*_2_ and *M*_3_ are performing the best. Only at checkpoint 16, at the very end of the trail, *M*_3_ outperforms the other models, while *M*_2_ is the best at checkpoints 7–15. For what concerns the observed and simulated number of marchers retiring at every checkpoint, the three models lead to similar distances, with only slightly lower distances for *M*_2_.

**Table 3:** Match and Kolmogorov-Smirnov distances between the observed and simulated edfs of the number of marchers retiring at every checkpoint.

Model	Match distance (-)	Kolmogorov-Smirnov distance (-)
*M*_1_	0.16	0.017
*M*_2_	0.15	0.015
*M*_3_	0.15	0.016

Considering these results, *M*_2_ was finally selected as optimal model describing the dynamics of the 100 km Dodentocht. This model was preferred to *M*_1_ since it leads to a better agreement between the observed and simulated passing times, while it was preferred to *M*_3_ because its construction is simpler and it leads at the same time to an equally good—and sometimes even better—agreement between the observed and simulated quantities.

## Model validation

Models are valuable only if they have been validated with respect to their purpose. The purpose of the spatially explicit marching model is to gain insight into the dynamics of the 100 km Dodentocht. Knowing the number of marchers passing by the checkpoints at every time instance is important for optimizing the operational efforts along the trail and is, as such, the most important aspect. However, since the simulated number depends on how the consecutive marching speeds of individual marchers are combined and on the number of marchers retiring at every checkpoint, also these two aspects are considered.

We resort to a predictive validation strategy [17]. In our setting, this involves assessing the performance of the spatially explicit model for a given edition (*i.e.* a given year) when the model is constructed from the dataset obtained by deleting all entries of that particular edition. Given the fact that the RFID dataset presented in the Section ‘Data collection and overview’ contains entries from five editions, such a validation should be done fivefold. For every edition, the Match and Kolmogorov-Smirnov distances between the observed and simulated edfs and the corresponding confidence intervals of these distances were calculated [17].

The simulations were set up as follows. The number of simulated marchers was equal to the number of marchers with a complete set of scans during the event under consideration. The percentage of male marchers and the conditional distributions ${\hat{F}}_{V_{s}|\;G,A,P}$, ${\hat{F}}_{V_{s}|\;G,P}$ and ${\hat{F}}_{V_{s}|\;G}$ were determined on the basis of the tracking data of the other editions. Per edition, 100 Monte Carlo simulations were performed.

**Fig 14:**
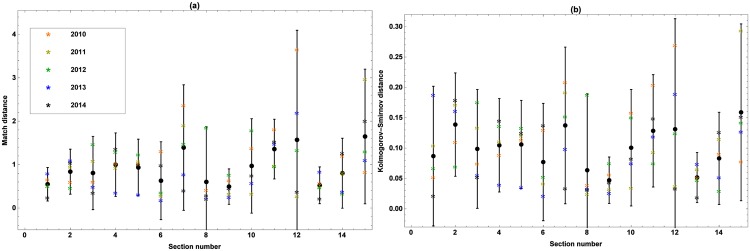
Match (a) and Kolmogorov-Smirnov (b) distances between the edfs of the observed and simulated marching speeds at every consecutive section. The average distances for the 5 validated editions are represented by colored asterisks, while the error bars represent the corresponding 95% confidence interval over all the editions.

**Fig 15:**
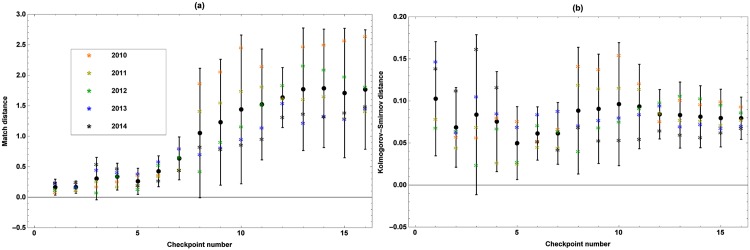
Match (a) and Kolmogorov-Smirnov (b) distances between the edfs of the observed and simulated number of marchers passing by at every checkpoint per 0.25 h for the 5 validated editions. The average distances for the 5 validated editions are represented by colored asterisks while the error bars represents the corresponding 95% confidence interval over all the editions.

The average Match and Kolmogorov-Smirnov distances between the edfs of the simulated and observed marching speeds at the consecutive sections and passing times along the consecutive checkpoints are shown in Figs 14 and 15, respectively. The asterisks represent the average distances for every edition, while the error bars represent the corresponding 95% confidence interval over all editions. The average Match and Kolmogorov-Smirnov distances per edition between the edfs of the simulated and observed number of marchers retiring at every checkpoint can be found in Table 4. When considering all editions, these distances are 0.35 and 0.04, respectively, while the 95% confidence intervals are [0.02, 0.68] and [0.01, 0.07].

**Table 4:** Match and Kolmogorov-Smirnov distances between the observed and simulated edfs of the number of marchers retiring at every checkpoint for every edition.

Validated edition	Average Match distance (-)	Average Kolmogorov-Smirnov distance (-)
2010	0.60	0.065
2011	0.40	0.045
2012	0.21	0.022
2013	0.37	0.037
2014	0.18	0.039

From Fig 14, it can be seen that the 95% confidence interval of the average Match distance is always located in [0, 4], while the one of the Kolmogorov-Smirnov distance is located in [0, 0.3]. Comparing this observation to the 95% confidence intervals obtained when an average edition is simulated on the basis of all available data (see Section Model selection, model *M*_2_), which were [0, 0.7] and [0, 0.06], respectively, it is apparent that the simulated marching speeds differ more from the recorded ones if the dynamics of a particular edition is simulated using the spatially explicit marching model that was constructed from the other editions. For what concerns the performance of the model across the editions, there is not a single one that stands out.

Considering the distances between the observed and simulated passing times, it can be seen from Fig 15 that the 95% confidence interval of the average Match distance is always located in [0, 3], while the one of the Kolmogorov-Smirnov distance always lies in [0, 0.2]. Comparing these intervals with the ones obtained when an average edition of the 100 km Dodentocht is simulated on the basis of all data (see Section Model selection, model *M*_2_), which are [0, 2.5] and [0, 0.11], respectively, it may be concluded that the passing times estimated for an edition on the basis of tracking data of other editions are equally well simulated as when all tracking data are used to build and validate the model. The average distances of edition 2010 are the largest.

For what concerns the distances between the distributions of the retiring dynamics, the average distances are approximately three times smaller when all data are used to build the model. Again, edition 2010 appears to be the most difficult one to simulate realistically (Table 4).

Considering the results for model *M*_2_ presented in Section Model selection and the validation results presented throughout this section, we conclude that the developed spatially explicit marching model enables us to reliably simulate the dynamics of an average 100 km Dodentocht edition, especially for what concerns the passing times as the predictive power for the latter is the highest.

## Scenario analysis

### Overview

After the starting signal of the 100 km Dodentocht, approximately 11 000 marchers start together. The first part of the trail passes through the narrow streets in the town center of Bornem, Belgium, where the marchers are motivated by their followers. It is so crowded along this part of the trail that the marchers almost have to progress in a flock, which is a situation that the organizers of the endurance event would like to overcome. For that reason, the organizing committee proposed three scenarios on the basis of their experience that are aimed at giving the marchers more space along the first part of the trail. Scenario 1 involves a starting location outside the town center, where the streets are wider, so that the marchers will be scattered more along the trail by the time they enter the town center. In Scenario 2, the starting marchers are divided into two or more groups that start at different locations. Eventually, these groups will meet one another when the marchers are more scattered along the trail. Finally, in Scenario 3 the start is organized in such a way that fewer people pass the starting line at once, which implies that the start will take longer than half an hour. This could be achieved by installing a bottleneck at the start.

In order to assess the effect of the proposed scenarios on the event dynamics, our spatially explicit model is adapted accordingly. Since these modifications imply changes to the starting conditions, the distribution of *V*_1_ needs to be adapted according to the scenario under consideration. Yet, for doing so, *V*_1_ has to be sampled without using the empirical joint distribution function ${\hat{F}}_{A\;,V_{1}|\;G}\left(a,v_{1}|g\right)$, but by relying on a distribution function describing *V*_1_ solely. The latter is conditioned on G, for which a value is chosen at the beginning of the simulation of a marcher. Since age and speed are correlated (cfr. Fig 9), both variables cannot be sampled independently. Here, we used a copula so that *V*_1_ can be sampled from its marginal distribution.

### The copula of starting speed and age

A bivariate copula *C*, *i.e.* a bivariate probability distribution function on [0, 1]^2^, was determined for describing the dependence between *V*_1_ and A. This copula was used together with the two marginal distribution functions of *V*_1_ and A, *i.e.* *F*_*V*_1__ and $F_{A}$, to estimate the joint distribution function [18]:

$$\begin{array}{c} F_{V_{1}A}\left(v_{1},a\right)=C\left(F_{V_{1}}\left(v_{1}\right),F_{A}\left(a\right)\right). \end{array}$$ (6)

By sampling a copula that links *V*_1_ and A, a value between 0 and 1 is obtained for both variables. Subsequently, the inverse transform method [13] was used to transform this value into a sample of *V*_1_ and A. Many parametric copula families have been reported in literature [19], of which we selected the Frank family [20] on the basis of the Kendall’s tau and the maximum likelihood [21]. The relevant copula parameters were 0.368 and 0.298, respectively for male and female marchers.

### The marginal distribution of the starting speed and its modifications

**Fig 16:**
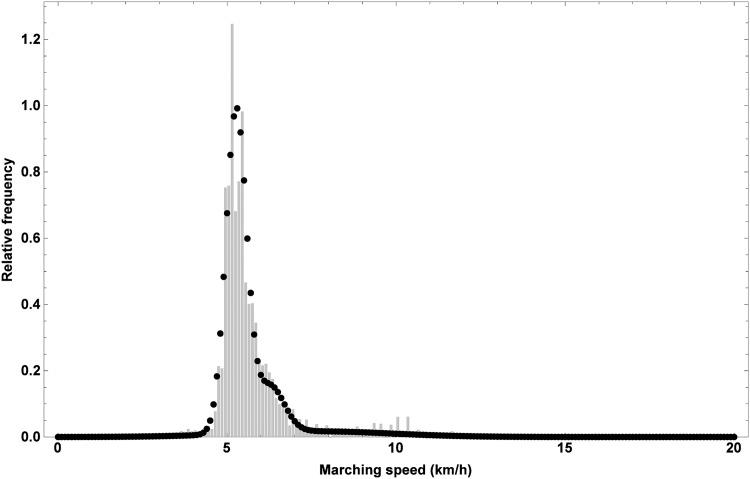
Relative frequency of the observed starting speed and the mixture distribution function that was fitted to the corresponding data for male marchers. The mixture distribution involves three normal distribution functions with parameters listed in Table 5.

In order to implement the outlined scenarios, the distribution of *V*_1_ has to be modified accordingly. To make meaningful adaptations, the distribution of *V*_1_ should be described analytically. On the basis of a preliminary analysis of the data, we chose to fit a mixture of three normal distribution functions M to the data for men. The parameters of the involved distributions were fit by using the maximum likelihood method in Mathematica (Version 9.0.1, Wolfram Research Inc., Champaign, US). They are listed in Table 5, while the fitted mixture distribution is shown in Fig 16, together with the relative frequencies of *V*_1_. Similarly, a mixture distribution for women was obtained. The first normal distribution of M, $N_{1}$, reflects the group of marchers of the 100 km Dodentocht, the third normal distribution, $N_{3}$, represents the group of joggers that gives rise to a right tail in the relative frequencies of the marching speeds and finally, the second normal distribution, $N_{2}$, relates to fast marchers whose characteristics are intermediate between those of joggers and marchers.

**Table 5:** Parameters defining the three normal distributions making up the mixture distribution describing *V*_1_.

Men				Women		
Denotation	Mean (km h^−1^)	Standard deviation (km h^−1^)	Weight	Mean (km h^−1^)	Standard deviation (km h^−1^)	Weight
$N_{1}$	5.27	0.31	75	5.21	0.26	72
$N_{2}$	6.26	0.43	15	5.96	0.33	14
$N_{3}$	7.57	2.32	10	6.42	2.17	14

Since the groups of joggers and fast marchers do not suffer from the crowd along the first part of the trail as they rapidly overtake the marchers, it may be anticipated that the proposed scenarios will mostly affect the dynamics of the group of marchers. As such, only the parameters of $N_{1}$ should be adapted when implementing the scenarios. Reducing the number of marchers per unit area along the first part of the trail will increase the starting speed of some marchers because they are less hindered by others, on the one hand, but might decrease the starting speed of others since they are no longer forced to move along with the crowd. In this way, marchers will be able to march at their preferred speed. The net effect will depend on the ratio between both types of marchers. Ultimately, these effects will imply changes to both the mean and the standard deviation of the distribution of the marchers’ marching speeds. For comprehensiveness, we first conduct a sensitivity analysis in order to assess the impact of a varying mean and standard deviation of $N_{1}$ on the event dynamics, after which we turn to an encoding of the scenarios under consideration.

#### Varying the mean starting speed of marchers

**Fig 17:**
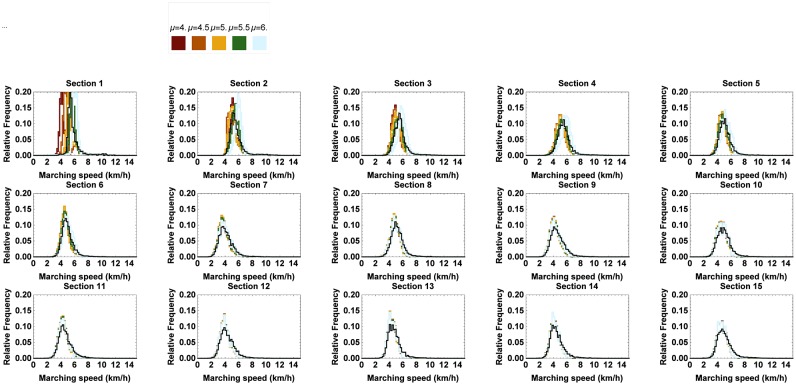
Relative frequency of the simulated marching speeds at the consecutive sections for different values of the mean starting speed of marchers. The 95% confidence interval of the simulated marching speeds when the mean of $N_{1}$ is successively varied between 4km h^−1^ and 6km h^−1^. The black line represents the benchmark situation.

**Fig 18:**
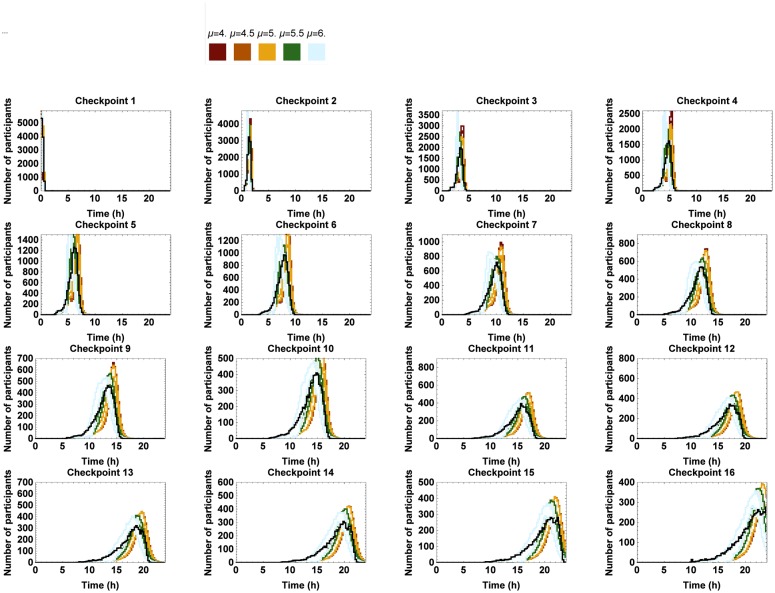
Number of marchers passing by at the consecutive checkpoints per 0.25 h for different values of the mean starting speed of marchers. The 95% confidence interval of the simulated passing times when the mean of $N_{1}$ is successively varied between 4km h^−1^ and 6km h^−1^. The black line represents the benchmark situation.

In order to examine the effect of a varying mean, the mean of $N_{1}$ was successively replaced by 4km h^−1^, 4.5km h^−1^, 5km h^−1^, 5.5km h^−1^ and 6km h^−1^ for both men and women, as opposed to the ones derived from the RFID data (cfr. Table 5). The relative frequency of the simulated marching speeds at every section and the passing times at the corresponding checkpoints can be found in Figs 17 and 18, respectively. One can see that the effect on the marching speed is the most pronounced along the first few sections and gently fades out along sections at greater distance from the start. If the average marching speed is higher than the one of the benchmark situation (*i.e.* as given by the RFID data), the difference between the distribution of the marching speeds of the marchers and the ones of the fast marchers and joggers becomes smaller.

The higher the mean marching speed, the earlier marchers pass the checkpoints. As the average marching speed increases, the maximum number of people passing per 0.25 h at the first three checkpoints becomes much higher than in the benchmark situation. This can be understood because the characteristics of the marchers gradually align with those of the fast marchers and joggers. Consequently, we conclude that it will become busier near the checkpoints when the average starting speed increases, which is the opposite of the situation pursued by the organizers.

#### Varying the standard deviation of the starting speed of marchers

**Fig 19:**
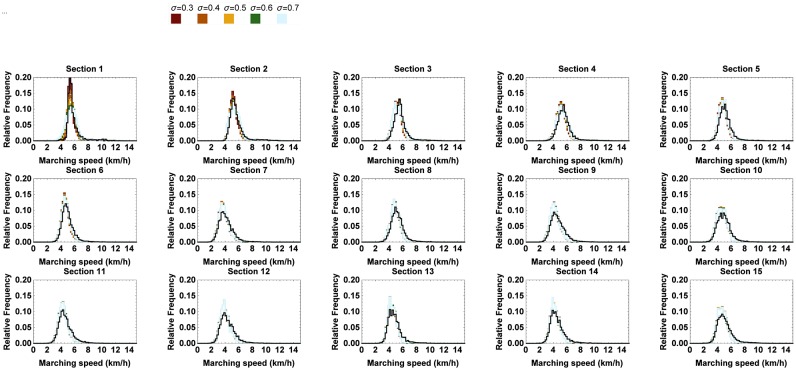
Relative frequency of the simulated marching speeds at every checkpoint for different values of the variation in starting speed of marchers. The 95% confidence interval of the simulated marching speeds where the standard deviation of $N_{1}$ is varied between 0.3km h^−1^ and 0.7km h^−1^. The black line represents the benchmark situation.

**Fig 20:**
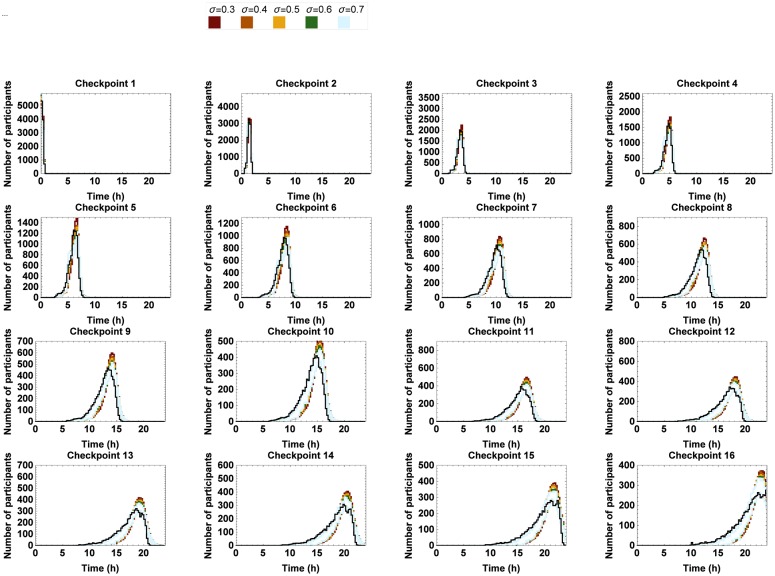
Number of marchers passing by at every checkpoint per 0.25 h for different values of the variation in starting speed of marchers. The 95% confidence interval of the simulated passing times where the standard deviation of $N_{1}$ is varied between 0.3km h^−1^ and 0.7km h^−1^. The black line represents the benchmark situation.

In order to examine the effect of a varying standard deviation, the standard deviation of $N_{1}$ was successively replaced by 0.3km h^−1^, 0.4km h^−1^, 0.5km h^−1^, 0.6km h^−1^ and 0.7km h^−1^ for both men and women, as opposed to the ones derived from the RFID data (cfr. Table 5). We chose to restrict our analysis to the effect of values greater than or equal to the ones of the data since the amount of variation in the starting speed was considered relatively low given the much more pronounced variability along sections further away from the start. The histograms of the simulated marching speeds along the subsequent sections and the passing times at the corresponding checkpoints can be found in Figs 19 and 20, respectively.

Apparently, increasing the standard deviation only affects the marching speeds along the first two sections of the trail. The shape of the histograms of the passing times closely resembles the shape of the distributions of the benchmark situation from checkpoint 3 on. Only at the first few checkpoints, it becomes a bit less busy. Increasing the standard deviation would thus give marchers more space to move freely along the first sections, as such improving the starting situation.

### Scenarios

#### Encoding

The scenarios outlined at the beginning of this section aim at reducing the density of marchers along the first part of the trail. For example, starting in two groups will reduce the density by 50% (Scenario 2). Furthermore, a density reduction of 10%, 25% and 50% could be the result of starting outside the town center, or by installing a bottleneck at the starting line (Scenarios 1 and 3). In order to assess the impact of these scenarios on the event dynamics, the parameters of $N_{1}$ were changed in order to reflect them appropriately.

The Kladek formula [22] expresses that the average marching speed increases with decreasing density. We used the revised formula from [23], which is valid for rush hour situations, to assess the effect of a decreasing density on the starting speed. From [23], it follows that 0.33m^−2^ is the critical density, *i.e.* the density that corresponds to the upper bound on the marchers’ density beyond which unconstrained marching becomes impossible. According to the revised Kladek formula, this density corresponds to a marching speed of 6.08km h^−1^ [23]. As such, people moving faster than 6.08km h^−1^ may be considered as marching freely. Considering the parameters in Table 5, we observe that the intended scenarios primarily affect the group of marchers described by $N_{1}$. Hence, it is justified to modify the parameters of this distribution only. In literature, it has been reported that the marching speed follows a normal distribution [22, 24], and that the standard deviation of freely marching individuals is 1.33km h^−1^ [24] but, to the best of our knowledge, there are no reports on the relation between the standard deviation and the density of the marchers.

The revised Kladek formula was used to estimate the density of the marchers at the start of the 100 km Dodentocht on the basis of the mean marching speeds given by $N_{1}$ for men and women (cfr. Table 5). We found a density of 0.72m^−2^ and 0.70m^−2^, respectively, resulting in an average density of 0.71m^−2^. Since this density is much higher than 0.33m^−2^, it is obvious that marchers cannot move freely along the first part of the trail of the 100 km Dodentocht.

Since the variability in starting speeds is relatively limited compared to the variability in marching speeds along sections further away from the start later, as most marchers did not retire along sections 2 and 3 and knowing that the speeds along those sections are still correlated with the starting speed (cfr. Section ‘Data analysis’), we studied the distribution parameters of the marching speed along sections 2 and 3 (*V*_2_ and *V*_3_). A mixture distribution of three normal distributions was also fitted to *V*_2_ and *V*_3_. For men, the first normal distribution of *V*_2_ and *V*_3_ has a standard deviation of 0.59km h^−1^ and 0.6km h^−1^, respectively. For women, these standard deviations are 0.5km h^−1^ and 0.51km h^−1^, respectively. Comparing these values with the ones in Table 5, it is obvious that the standard deviation along the first section is nearly half of the ones along sections 2 and 3. As such, the average standard deviation of the distributions of *V*_2_ and *V*_3_ was used (*i.e.* 0.60km h^−1^ for men and 0.51km h^−1^ for women) to replace the standard deviation of $N_{1}$.

When running simulations for scenarios involving an increased average starting speed, marchers are expected to cross the starting line earlier than in the benchmark situation (cfr. Fig 11), as they align with fast marchers and joggers. Consequently, all marchers would have crossed the starting line in a shorter time span, which means that the density of marchers along the first section would again increase. This should be avoided by managing the number of people passing the starting line in such a way that the duration of the starting procedure does not change. In Scenario 3, however, the duration of the start should be longer as fewer people are allowed to pass the starting line at once compared to the benchmark situation. For that purpose, the sampled passing times were rescaled.

#### Analysis

The impact of the scenarios on the event dynamics and operational efforts was assessed by means of in silico experiments. More precisely, 100 events were simulated, with 10 000 marchers each, per scenario. The obtained in silico event dynamics was compared with the benchmark situation.

**Scenario 1** Scenario 1 involves a start that is located outside the center of Bornem, Belgium. Obviously, to what extent the density of the marchers is reduced, depends on the amount of additional space that the wider streets outside the town center bring along. For that reason, we evaluated the effect of a reduction of the marcher density at the start by 10%, 25% and 50%. Using the revised Kladek formula, these reductions correspond to an average starting speed of 5.44km h^−1^, 5.69km h^−1^ and 5.99km h^−1^, respectively. Besides, the standard deviation of $N_{1}$ was chosen to be 0.60km h^−1^ for men and 0.51km h^−1^ for women. Decreasing the density at the start even further would not change the distribution parameters substantially, since the marchers can almost move freely when it is reduced by 50%. In agreement with the sensitivity analysis presented in Section ‘The marginal distribution of the starting speed and its modifications’, these reductions only affect the marching speeds along the first two sections, while the passing times are affected between checkpoints 2 and 6. Moreover, the maximum frequency of marchers passing the checkpoints per 0.25 h occurs around the same time as in the benchmark situation, but the average maximum number of people passing per 0.25 h at checkpoints 2 to 3 is higher than in the benchmark situation. This increase becomes more pronounced as the density decreases more, and can also be observed at checkpoints farther away from the start (Table 6). At those checkpoints, the organization should deploy more resources.

**Table 6:** Average difference between the observed and simulated maximum number of marchers per 0.25 h. The average difference between the observed and simulated maximum number of marchers per 0.25 h with a density reduction of 10%, 25% and 50% at the start, obtained by means of wider streets. The maximum number of people passing per 0.25 h interval in the benchmark situation is given in the first row.

Checkpoint ID	1	2	3	4	5	6	7	8	9	10	11	12	13	14	15	16
Benchmark situation	5332	3228	1992	1615	1262	971	721	537	464	410	394	344	322	308	281	276
Density reduction of 10%	-226	482	330	31	-49	-46	-43	5	27	29	14	29	33	36	48	41
Density reduction of 25%	-226	639	539	260	79	-34	-87	-37	-8	1	-10	11	19	23	39	31
Density reduction of 50%	-222	1031	805	517	327	109	-35	-47	-30	-12	-22	2	12	15	32	23

This observation can be explained by the fact that the characteristics of the marchers align with those of joggers and fast marchers, as such jeopardizing the mitigating effect of wider streets. At checkpoint 2, for example, the maximum number of marchers registered per 0.25 h increases by 15%, 20% and 32% as compared to the benchmark situation if the density is reduced by 10%, 25% and 50%, respectively. Assuming that the streets are still 10%, 25% or 50% wider at checkpoint 2, this implies a net density increase of 5%, a decrease of 5% and a decrease of 18%, respectively, at that particular checkpoint, which implies that the streets should be at least 25% wider in order to have a beneficial effect on the marcher density along the trail.

The passage of the event through Bornem is considered as a true town fest. The presence of the marchers and their supporters has a substantial commercial value for local businesses in Bornem. Therefore, it is important that the participants pass as early as possible through the town center so that their supporters visit the town shops, restaurants and pubs. In the benchmark situation, at most 5332 marchers pass at checkpoint 1 per 0.25 h, and a density reduction of at least 50% is needed to ensure that the marchers can move freely along that part of the trail (cfr. Section ‘Encoding’). Hence, at most 2666 may pass per 0.25 h by the checkpoint located in the town center. Consulting Table 6, we may conclude that, despite the obtained density reduction, the maximum number of people passing by checkpoint 3 (*i.e.* 17.6 km) in a 0.25 h interval has decreased to such an extent that it may be located in the town center, while still not causing any discomfort for the marchers.

Scenario 1 implies that a new route has to be laid out taking into account the findings above. As such, this implies that one or more new locations for checkpoints have to be identified and, since the start would be outside the town center, additional drinking and eating stands have to be installed on top of to the usual equipment. Consequently, introducing this scenario would require organizational efforts. Still, once the structural changes have been introduced, they can remain unchanged for several years. Moreover, the efforts have to be made prior to the event, while there would not be an extra work load during the event since opening hours and number of participants passing by at the consecutive checkpoints would change only slightly. Nevertheless, since marchers would only pass through the town center after 17.6 km, this scenario jeopardizes the revenues of the local businesses and the popularity among the locals. A starting location at the outskirts of the town so that the marchers leave the town into a wide street, might be a compromise since they and their followers could then still linger around town at the beginning of the event.

**Scenario 2** In Scenario 2, the marchers are divided into two or more groups that start at different locations. Eventually, these groups will meet when the marchers are already more scattered along the trail. Considering two groups, a start that takes up to half an hour and a street design as in the benchmark situation, it follows that the density at the start reduces with 50%, so that the simulation results boil down to the ones for Scenario 1. Yet, the number of people passing per 0.25 h as reported in Table 6 should be divided by two. Doing so, it may be concluded that the two groups ideally merge at a distance of about 17.6 km (checkpoint 3) because the maximum number of people passing by is lower than 2666, as such allowing free movement. Considering even more groups would not have a much larger effect because the marchers can move almost freely as soon as they are divided into two groups.

Scenario 2 would involve extra work for the organizers both prior to and during the event. An alternative, parallel route for the first 17.6 km has to be found, which then has to be made free of traffic, equipped with toilets, etc. Furthermore, an extra start and second checkpoint have to be chosen. On the other hand, however, less people pass by these checkpoints, so less volunteers have to be present per checkpoint. In order to avoid disadvantages for the local businesses (cfr. Scenario 1), the organizers should install both starting locations in the town center.

**Scenario 3** The third scenario aims at reducing the density by installing a bottleneck at the start in order to limit the number of marchers that can pass per unit of time. Again, we consider a reduction of the density at the start by 10%, 25% and 50%. As opposed to Scenarios 1 and 2, the start will take longer, which was mimicked by rescaling the starting times drawn from the distribution function of the experimental starting times, *i.e.* by dividing them by 0.9, 0.75 and 0.5, respectively. The maximum number of people passing per 0.25 h at checkpoint 1 is lower, irrespective of the density reduction, as compared to the benchmark situation, with the lowest frequencies occurring for the highest density reduction (Table 7).

**Table 7:** Average difference between the observed and simulated maximum number of marchers per 0.25 h. The average difference between the observed and simulated maximum number of marchers per 0.25 h with a density reduction of 10%, 25% and 50% at the start, obtained by means of a longer duration of the start. The maximum number of people passing per 0.25 h interval in the benchmark situation is given in the first row.

Checkpoint ID	1	2	3	4	5	6	7	8	9	10	11	12	13	14	15	16
Benchmark situation	5332	3228	1992	1615	1262	971	721	537	464	410	394	344	322	308	281	276
Density reduction of 10%	-613	244	248	28	-56	-49	-49	4	23	29	14	27	30	33	47	40
Density reduction of 25%	-1180	80	303	152	44	-41	-89	-42	-10	1	-9	10	20	22	38	31
Density reduction of 50%	-2209	-395	-99	7	49	6	-61	-54	-36	-19	-32	-6	3	8	28	22

Similar to the alignment of marchers with fast marchers and joggers observed in Scenario 1, the mitigating effect of a longer duration of the start is partly compensated by the higher average marching speed. Apparently, the density should be reduced by 50% in order to arrive at lower densities at checkpoints 2 and 3.

The longer duration of the start implies that the closing times of the checkpoints should be postponed, while the opening times should stay the same so their total operating time would increase. Based on the in silico results, we concluded that checkpoints should be opened for an extra one and a half hour along the first part of the trail and for one extra hour near the end of the trail. The flow of people would be more gradual, but the peaks in the number of participants would remain high from checkpoint 4 on, so we expect that the operational efforts will be somewhat higher.

## Discussion

In this paper, we presented a validated spatially explicit model for simulating the endurance marching event ‘the 100 km Dodentocht’. To the best of our knowledge, this work is the first of its kind aiming at building a spatially explicit model that mimics the dynamics of such an endurance event on the basis of RFID tracking data. The model is data driven because it relies on the patterns that are uncovered in the RFID dataset and finally encoded in terms of conditional distribution functions and copulas.

The proposed model is able to mimic the dynamics of the 100 km Dodentocht realistically, which comes forward by the fact that the relative frequencies of the observed and simulated marching speeds along the consecutive sections of the trail are similar (cfr. Figs 12 and 14). For what concerns the number of people passing per 0.25 h at the consecutive checkpoints, the corresponding histograms have a similar shape, but from checkpoint 7 on the peaks are overestimated, while the tails towards the left (earlier passing times) are underestimated (cfr. Figs 13 and 15). This is caused by the fact that pauses cannot be identified using the RFID data. These pauses make that there are sudden drops in the observed marching speeds that cannot be grasped by the spatially explicit marching model. More explanatory variables and information about the resting behavior are needed to incorporate this correctly. The spatially explicit model may be used to assess the possible effects of changes to the trail, the organization and the characteristics of the marchers that have an impact on the marching speed and the resulting passing times. Often, it is crucial to know the impact of such modifications on the event dynamics before implementing them, but aside from relying on a mathematical model, there is no way to accomplish this. In this paper, we examined the effects of structural changes to the trail (Scenario 1), and the starting procedure (Scenarios 2 and 3) attempting at a decrease of the marchers’ density. Based on our simulation results, we may conclude that a decrease of the density at the start does not necessarily induce such a decrease at subsequent checkpoints. A density reduction at the start of at least 50% implies lower densities at all subsequent sections, irrespective of the specific measure under consideration. As such, it is important to estimate the density reduction that matches the measure under consideration in order to distribute the resources efficiently over the trail.

The developed spatially explicit marching model could also be used to assess the effects of changes in the age distribution of the marchers or the proportion of female marchers. This is an especially interesting perspective given the changing demography in Belgium and many other countries.

Similar to the 100 km Dodentocht, most endurance events have a predefined route with several checkpoints and make use of RFID tracking systems to follow their participants. Therefore, the presented framework can be used for analyzing and improving other such events. Yet, sufficient tracking data should be available in order to grasp the variability among the participants, and finally build reliable edfs that determine the speeds on the basis of some known features. A data analysis revealing the features influencing the speed of the participants should always be performed prior to the determination of the conditional edfs.
